# Supplementary material for: Brain methylome remodeling selectively regulates neuronal activity genes linking to emotional behaviors in mice exposed to maternal immune activation
Source: Nat Commun. 2023 Nov 29;14:7829. doi: 10.1038/s41467-023-43497-4 (PMC10687003; doi:10.1038/s41467-023-43497-4)
Supplement: Supplementary file 1 — Supplementary Information [file 41467_2023_43497_MOESM1_ESM.pdf]

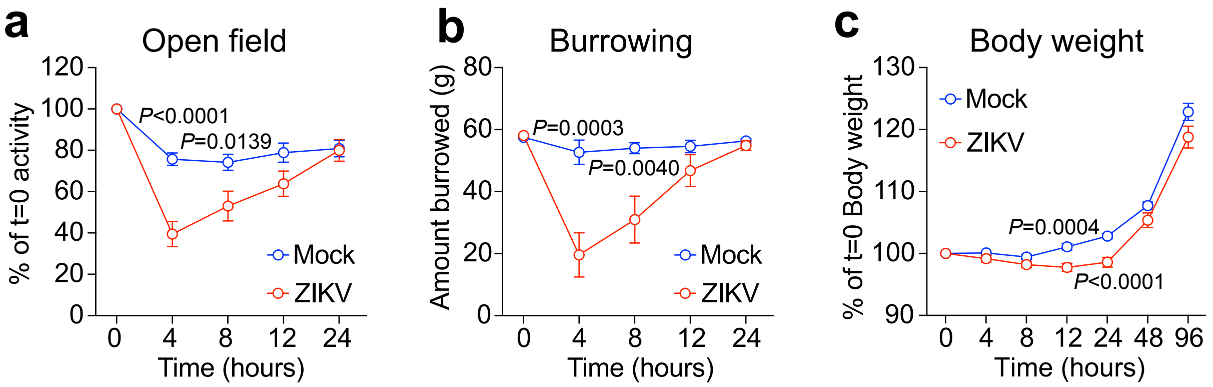

Supplementary Fig. 1: Pregnant female mice infected with ZIKV at E12.5 exhibit temporal sickness behaviors.

Relative distance traveled in the open field (**a**), amount of bedding burrowed (**b**) and relative body weight (**c**) measured before injections (0 h) and at 4, 8, 12 and 24 h post-injection. **a**, mock n = 14; ZIKV n = 13 mice; **b**, mock n = 14; ZIKV n = 12 mice; **c**, mock n = 16; ZIKV n = 18 mice. All data are presented as mean values ± SEM. *P* values were calculated by two-tailed unpaired t test. Source data are provided as a Source Data file.

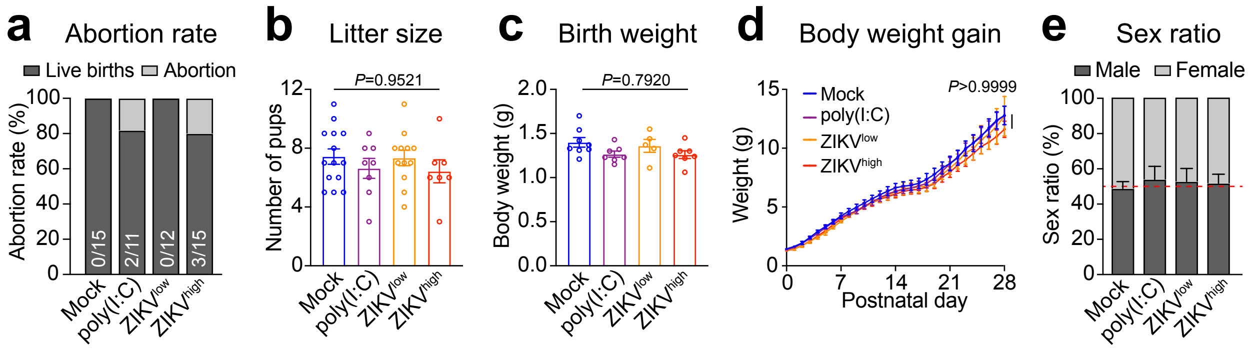

Supplementary Fig. 2: Pregnancy outcomes in the studies of maternal immune activation induced by poly(I:C) and low/high dose of ZIKV on gestation day 12.5.

a The spontaneous abortion rate of dams in each treatment group. The numbers in the bar plots represent the number of dams with spontaneous abortion out of the total number of dams recorded. Mock n=15; poly(I:C) n=11; $ZIKV^{low}$ n=12; $ZIKV^{high}$ n=15 dams. **b** The litter size delivered by dams that successfully maintained pregnancy. Mock n=14; poly(I:C) n=8; $ZIKV^{low}$ n=12; $ZIKV^{high}$ n=7 litters. **c** The average birth weight per litter delivered by dams in each treatment group. Mock n=8; poly(I:C) n=7; $ZIKV^{low}$ n=5; $ZIKV^{high}$ n=7 litters. **d** The average body weight of each litter delivered by dams in each treatment group from postnatal day 0 to 28. Mock n=7; poly(I:C) n=6; $ZIKV^{low}$ n=4; $ZIKV^{high}$ n=9 litters. **e** The sex ratio of offspring born to dams that successfully maintained pregnancy. Mock n=11; poly(I:C) n=9; $ZIKV^{low}$ n=9; $ZIKV^{high}$ n=9 litters. All data are presented as mean values ± SEM. *P* values were calculated by Welch and Brown-Forsy one-way ANOVA test (**b** and **c**) and two-way ANOVA test (**d**). Source data are provided as a Source Data file.

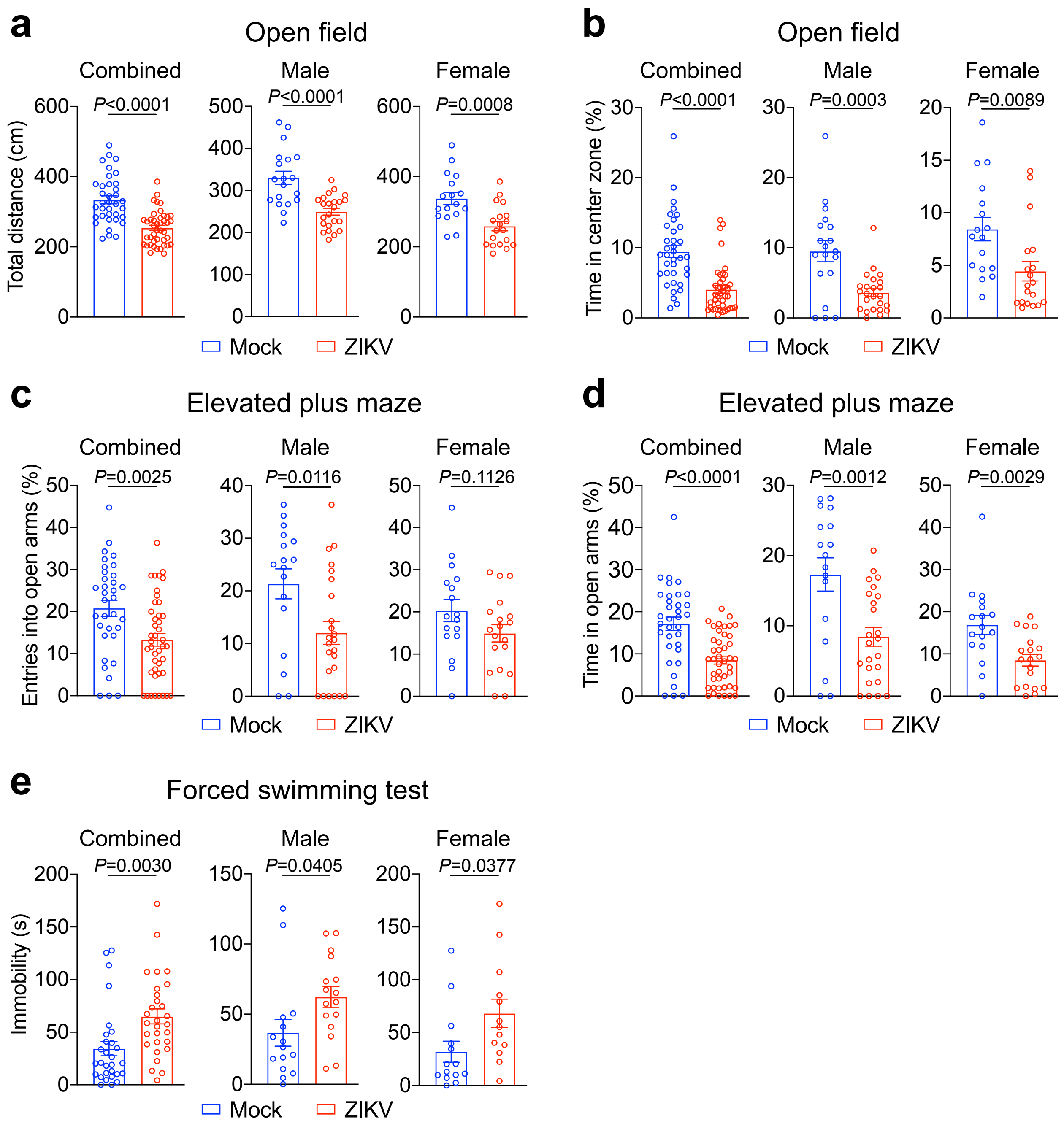

a
Open field
Combined
Male
Female
P<0.0001
P<0.0001
P=0.0008
Total distance (cm)
b
Open field
Combined
Male
Female
P<0.0001
P=0.0003
P=0.0089
Time in center zone (%)
Mock
ZIKV
c
Elevated plus maze
Combined
Male
Female
P=0.0025
P=0.0116
P=0.1126
Entries into open arms (%)
d
Elevated plus maze
Combined
Male
Female
P<0.0001
P=0.0012
P=0.0029
Time in open arms (%)
Mock
ZIKV
e
Forced swimming test
Combined
Male
Female
P=0.0030
P=0.0405
P=0.0377
Immobility (s)
Mock
ZIKV

Supplementary Fig. 3: The anxiety- and depression-like behaviors of offspring exposed to maternal ZIKV infection are not sex-biased.

a Total distance traveled in the open field test. Combined-Mock n = 36; Combined-ZIKV n = 42; Male-Mock n=19; Male-ZIKV n=23; Female-Mock n=17; Female-ZIKV n=19 mice. **b** Percentage of time spent in the center area in the open field test. Combined-Mock n=36; Combined-ZIKV n=42; Male-Mock n=19; Male-ZIKV n=23; Female-Mock n=17; Female-ZIKV n=19 mice. **c** Percentage of number of entries into open arms during elevated plus maze test. Combined-Mock n=34; Combined-ZIKV n=43; Male-Mock n=17; Male-ZIKV n=24; Female-Mock n=17; Female-ZIKV n=19 mice. **d** Percentage of time spent in the open arms during elevated plus maze test. Combined-Mock n=34; Combined-ZIKV n=43; Male-Mock n=17; Male-ZIKV n=24; Female-Mock n=17; Female-ZIKV n=19 mice. **e** Immobility time in the forced swimming test. Combined-Mock n=29; Combined-ZIKV n=29; Male-Mock n=15; Male-ZIKV n=16; Female-Mock n=14; Female-ZIKV n=13 mice. All data are presented as mean values ± SEM. *P* values were calculated by two-tailed unpaired t test. Source data are provided as a Source Data file.

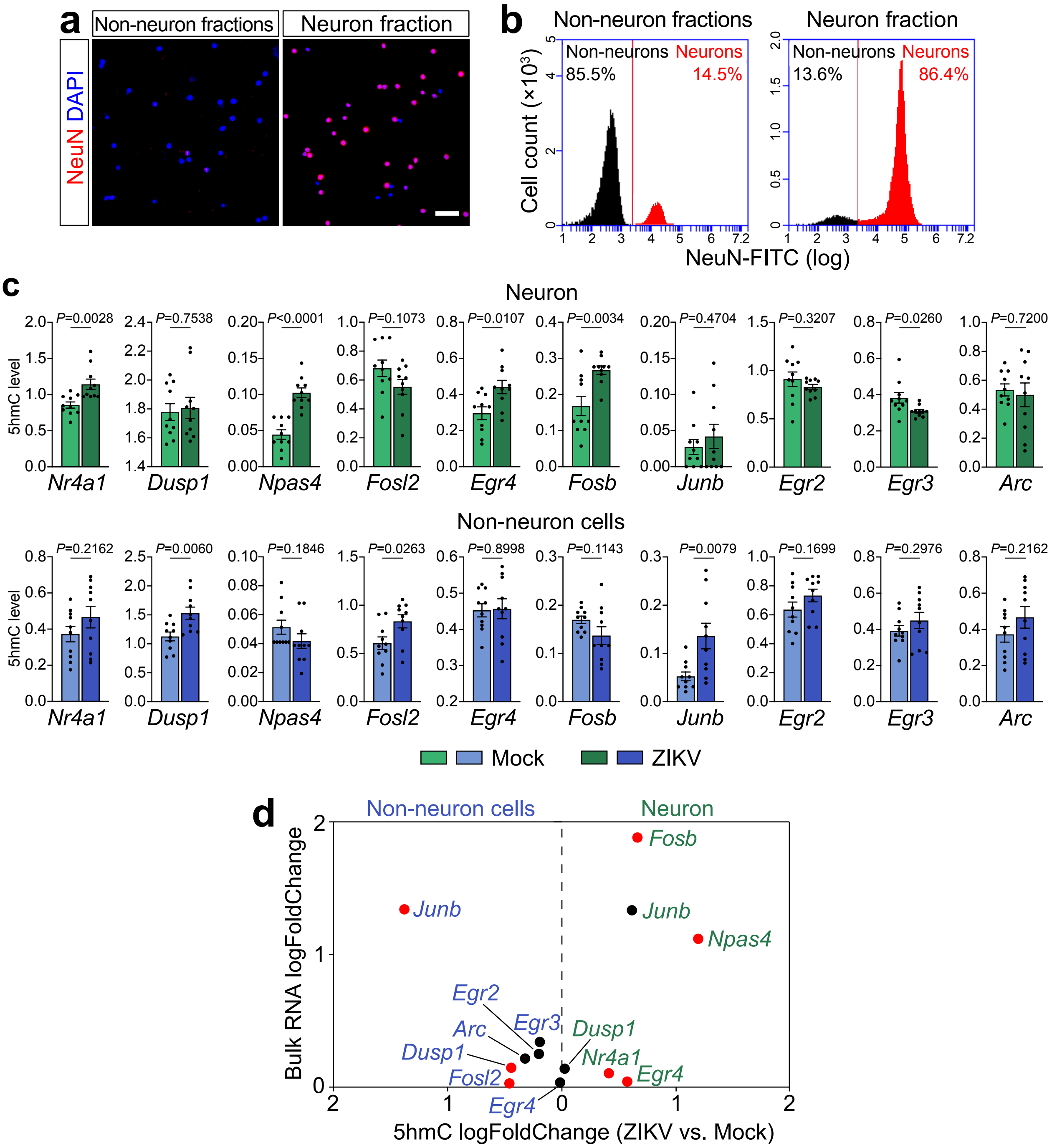
a
Non-neuron fractions
Neuron fraction
NeuN DAPI
b
Non-neuron fractions
Neuron fraction
Non-neurons
85.5%
Neurons
14.5%
Non-neurons
13.6%
Neurons
86.4%
Cell count (×10³)
NeuN-FITC (log)
c
Neuron
5hmC level
P=0.0028
P=0.7538
P<0.0001
P=0.1073
P=0.0107
P=0.0034
P=0.4704
P=0.3207
P=0.0260
P=0.7200
Nr4a1
Dusp1
Npas4
Fosl2
Egr4
Fosb
Junb
Egr2
Egr3
Arc
Non-neuron cells
5hmC level
P=0.2162
P=0.0060
P=0.1846
P=0.0263
P=0.8998
P=0.1143
P=0.0079
P=0.1699
P=0.2976
P=0.2162
Nr4a1
Dusp1
Npas4
Fosl2
Egr4
Fosb
Junb
Egr2
Egr3
Arc
Mock
ZIKV
d
Non-neuron cells
Neuron
Bulk RNA logFoldChange
5hmC logFoldChange (ZIKV vs. Mock)
Fosb
Junb
Junb
Npas4
Egr2
Egr3
Arc
Dusp1
Dusp1
Nr4a1
Egr4
Fosl2
Egr4

Supplementary Fig. 4: 5hmC alteration in IEGs of neuron and non-neuron cells isolated from ZIKV offspring mice.

a Representative images of $NeuN^+$ cells in the isolated non-neuronal (left) and neuronal fractions (right). Three independent experiments was repeated with similar results. Scale bar, 50 µm. **b** Histograms of NeuN-FITC fluorescence intensity in non-neuronal (left panel) or neuronal (right panel) isolated fractions. **c** Gene body 5hmC changes upon ZIKV infection in IEGs of neuronal (upper panel, green) and non-neuronal cells (lower panel, blue) measured by 5hmC-seq. n=10 per group. All data are presented as mean values ± SEM. *P* values were calculated by two-tailed unpaired t test. **d** Log2Fold Change of upregulated IEGs in bulk PFC (Y-axis), gene body 5hmC in isolated non-neuronal (left, blue text, 5hmC-seq) and neuronal (right, green text, 5hmC-seq) fractions (Y-axis). Red dots highlight upregulated IEGs with significant gene body 5hmC change ($P<0.05$) in neuronal or non-neuronal cells. Source data are provided as a Source Data file.

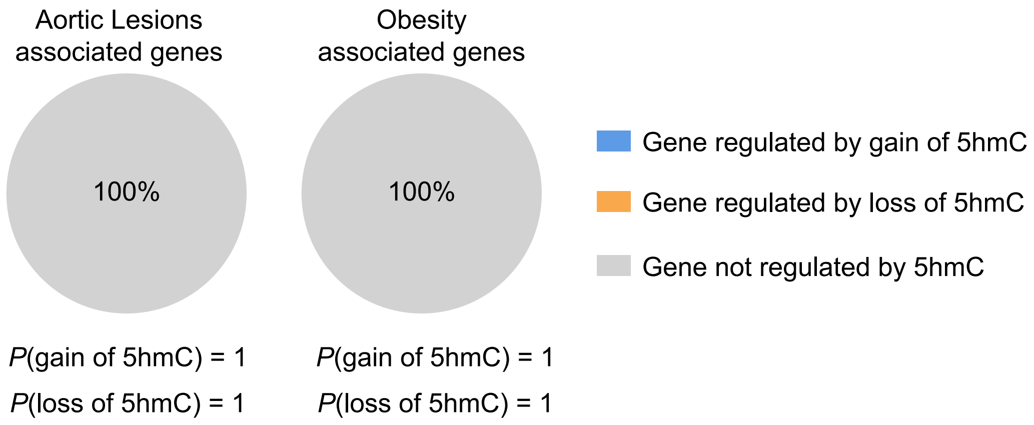

Supplementary Fig. 5: Correlation studies of dynamic 5hmC markers in ZIKV offspring mice.

Dynamic 5hmC-marked genes in ZIKV offspring mice do not overlap with genes related to aortic lesions and obesity. *P*-values are indicated in the figure and were calculated using binomial tests.

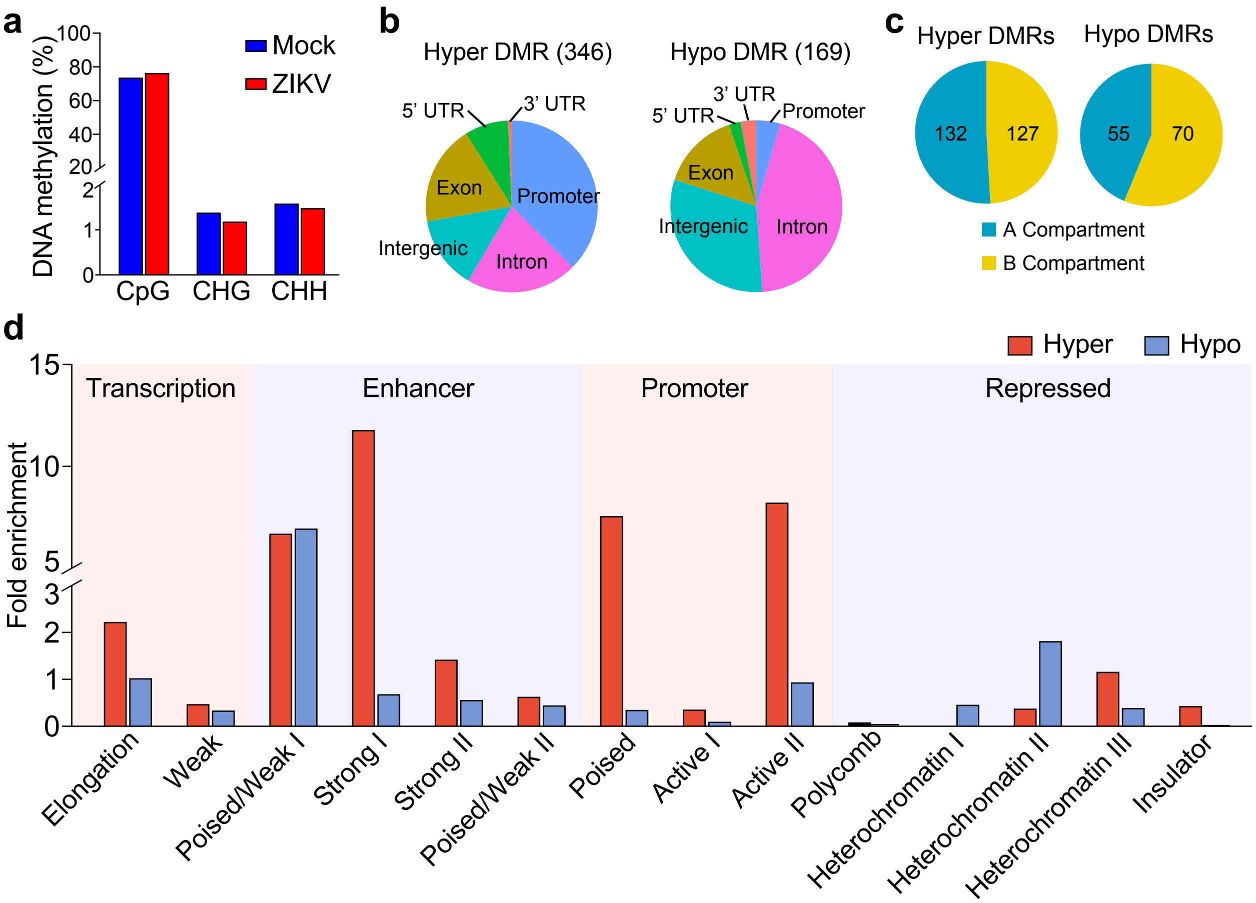

Supplementary Fig. 6: Characterization of DNA methylation in ZIKV offspring brains.

a The percentage of the genome-wide methylation level of CpG, CHG and CHH pattern in the cortex of Mock and ZIKV groups. **b** Genomic distribution feature for DMR in ZIKV-exposed offspring mice. **c** The distribution of Hyper DMRs (left) and Hypo DMRs (right) in A/B compartments. **d** The ChromHMM enrichment analysis for the Hyper and Hypo DMRs. Source data are provided as a Source Data file.

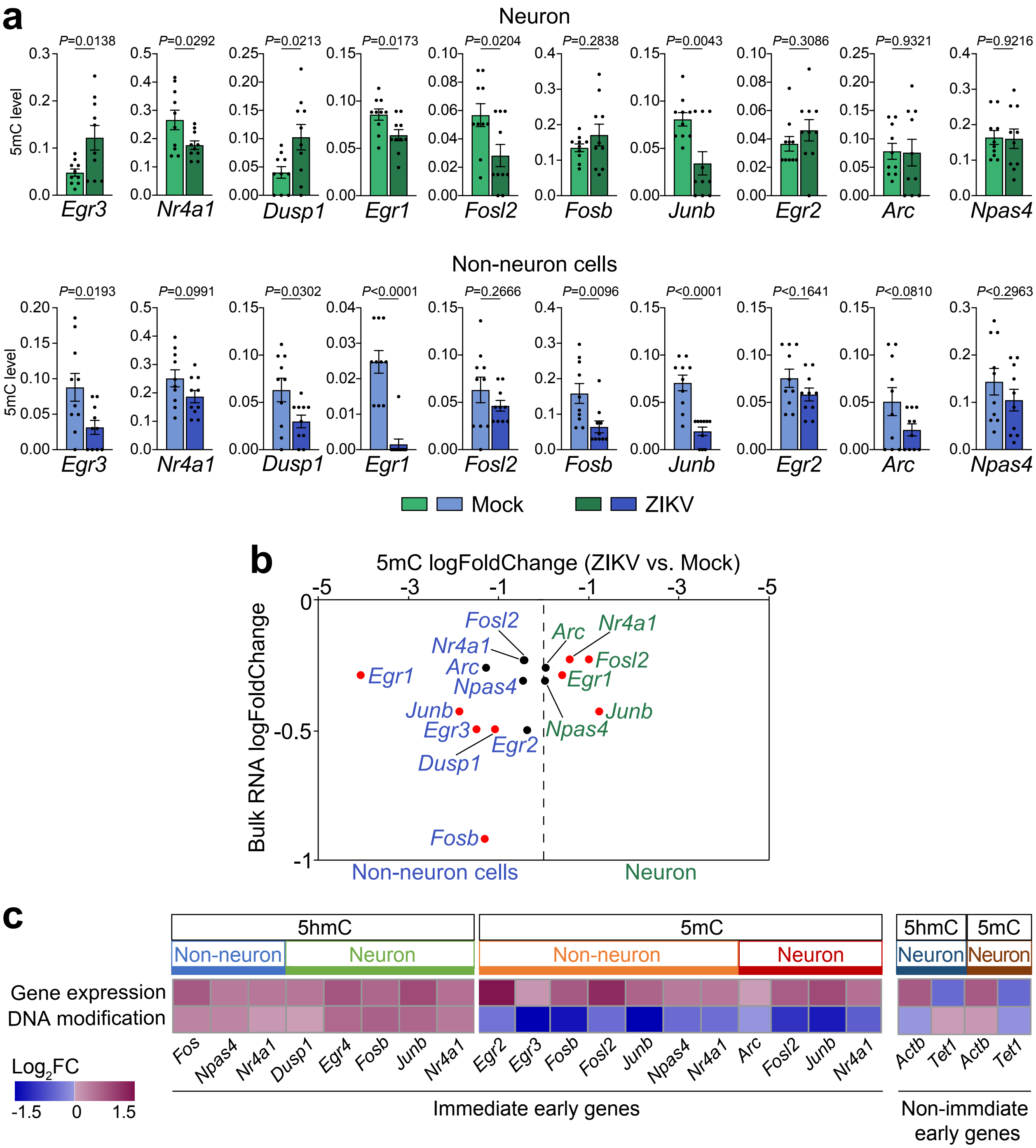
a
Neuron
5mC level
P=0.0138
P=0.0292
P=0.0213
P=0.0173
P=0.0204
P=0.2838
P=0.0043
P=0.3086
P=0.9321
P=0.9216
Egr3
Nr4a1
Dusp1
Egr1
Fosl2
Fosb
Junb
Egr2
Arc
Npas4
Non-neuron cells
P=0.0193
P=0.0991
P=0.0302
P<0.0001
P=0.2666
P=0.0096
P<0.0001
P<0.1641
P<0.0810
P<0.2963
Mock
ZIKV
b
5mC logFoldChange (ZIKV vs. Mock)
Bulk RNA logFoldChange
Non-neuron cells
Neuron
c
5hmC
5mC
Non-neuron
Neuron
Gene expression
DNA modification
Log2FC
-1.5
0
1.5
Fos
Npas4
Nr4a1
Dusp1
Egr4
Fosb
Junb
Egr2
Egr3
Fosl2
Arc
Actb
Tet1
Immediate early genes
Non-immdiate early genes

Supplementary Fig. 7: 5mC alteration in IEGs of neuron and non-neuron cells isolated from ZIKV offspring mice.

a Promoter 5mC changes upon ZIKV infection in IEGs of neuron (upper panel, green) and non-neuron cells (lower panel, blue) measured by MeDIP-seq. n=10 per group. Data are presented as mean values ± SEM. *P* values were calculated by two-tailed unpaired t test. **b** Log2Fold Change of upregulated IEGs in ZIKV bulk PFC (Y-axis), promoter 5mC upon ZIKV infection in isolated non-neuron (left, blue text, MeDIP-seq) and neuron (right, green text, MeDIP-seq) fractions (X-axis). Red dots highlight upregulated IEGs with significant decreases in promoter 5mC ($P<0.05$) in neuron or non-neuron cells. **c** Heatmap showing that IEG upregulation from single nuclear RNA-seq (snRNA-seq) data correlates with increased 5hmC and decreased 5mC from $NeuN^+$ or $NeuN^-$ cell populations. In contrast, non-IEG genes *Actb* and *Tet1* do not show such correlation. Source data are provided as a Source Data file.

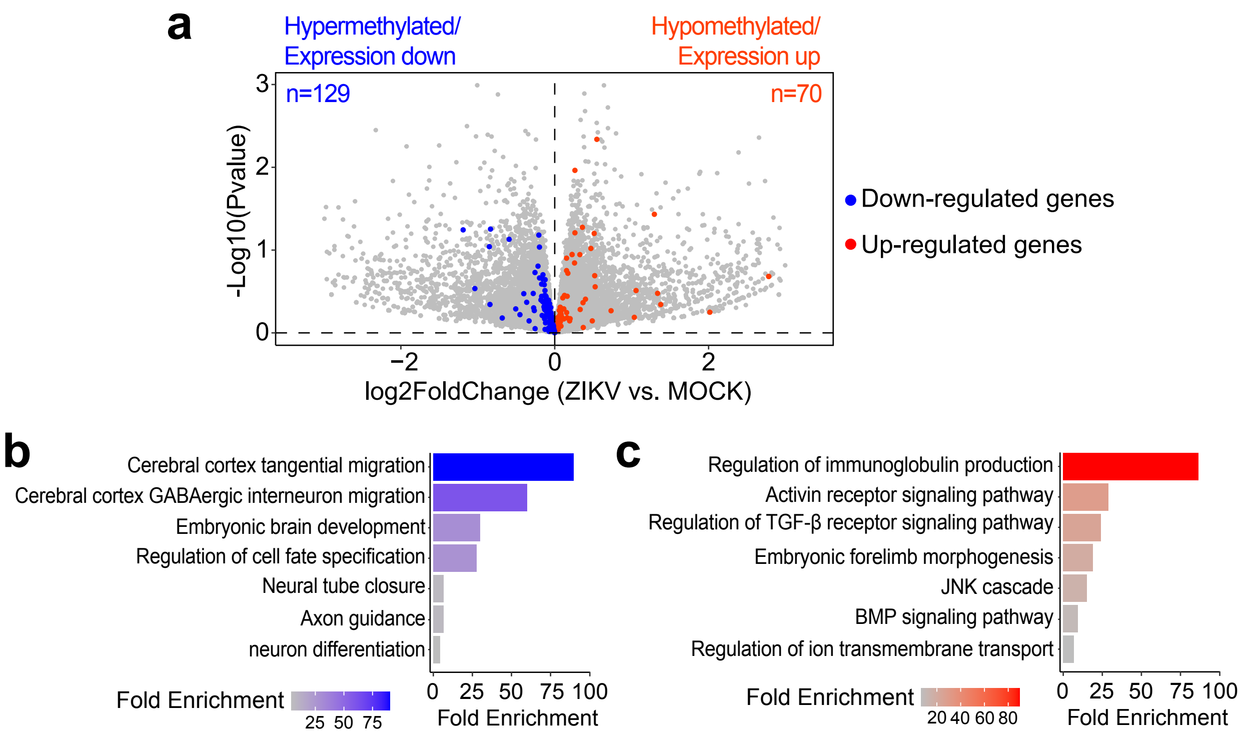

Supplementary Fig. 8: Gene expression changes with concomitated change of 5mC alteration in the PFC of offspring mice exposed to maternal ZIKV infection.

a Gene expression change in PFC of offspring mice exposed to maternal ZIKV infection. Down-regulated genes with hypermethylated features are highlighted in blue; upregulated genes with hypomethylated features are highlighted in red. RNA-seq log2FoldChange and P-value were plotted, n=4 biological replicates. DEseq2 were used for 5hmC differential analysis, multiple test adjustment was performed by p.adjust function, method fdr in R, significant 5hmC change was defined by FDR (False Discorvery Rate) <0.05, n=3 biological replicates. **b-c** Functional enrichment analysis of down (**b**) or up (**c**) regulated genes.

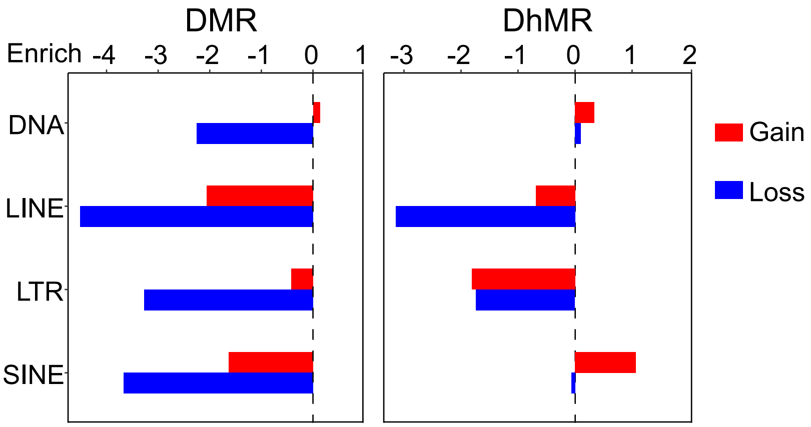

Supplementary Fig. 9: Enrichment analysis of DMR and DhMR identified from the PFC of offspring mice exposed to maternal ZIKV infection to TE families.

Log2 (number of observed regions / number of expected regions) calculated by HOMER were plotted for transposable elements including DNA, LINE, LTR and SINE for both differential methylation regions (DMR, left panel) and differential hydroxymethylation regions (DhMR, right panel). Red and blue bars represent gain and loss of methylated or hydroxymethylated regions in the PFC of offspring mice exposed to maternal ZIKV infection, respectively.

Supplementary Table 1 Sequences of primers used for real-time qPCR analysis (5'-3')

Figure	Primer	Sequence (5'-3')
Fig. 2c	Egr1-qPCR-forward	CGAGTTATCCCAGCCAAACG
Fig. 2c	Egr1-qPCR-reverse	GAAGACGATGAAGCAGCTGG
Fig. 2c	Egr2-qPCR-forward	CCATTGACCCACAGTACCCT
Fig. 2c	Egr2-qPCR-reverse	ACAGCCCGAATAAGGAGGAG
Fig. 2c	Egr3-qPCR-forward	GCAACAAGACCGTGACCTAC
Fig. 2c	Egr3-qPCR-reverse	GTTGGAATAAGGAGGCAGCG
Fig. 2c	Egr4-qPCR-forward	CTTCTTCATCCAGGCGGTTC
Fig. 2c	Egr4-qPCR-reverse	GATCTGGGGAGTAGAGGTCC
Fig. 2c	Npas4-qPCR-forward	TGAAGACATTGTGGCAGCAC
Fig. 2c	Npas4-qPCR-reverse	TGGTCAGCAGGGTCAATGAT
Fig. 2c	Fos-qPCR-forward	CCCTGGATTTGACTGGAGGT
Fig. 2c	Fos-qPCR-reverse	GCCTAGATGATGCCGGAAAC
Fig. 2c	Arc-qPCR-forward	ATACCGTTAGCCCCTATGCC
Fig. 2c	Arc-qPCR-reverse	GTGGTTCTGGATCTGGGACA
Fig. 2c	Fosb-qPCR-forward	AGGAACCAGCTACTCAACCC
Fig. 2c	Fosb-qPCR-reverse	AAGTCGATCTGTCAGCTCCC
Fig. 2c	Fosl2-qPCR-forward	CACCTCCATGTCCAATCCCT
Fig. 2c	Fosl2-qPCR-reverse	GACAGCTGCTCATCTCTCCT
Fig. 2c	Nr4a1-qPCR-forward	GACTTGCTCTCTGGTTCCCT
Fig. 2c	Nr4a1-qPCR-reverse	AGAAGGCCAGGATGTTGTCA
Fig. 2c	Junb-qPCR-forward	GCAGCTACTTTTCGGGTCAG
Fig. 2c	Junb-qPCR-reverse	CGTCCAGGGCTTTGACAAAA
Fig. 2c	Dusp1-qPCR-forward	CGCCTTGATCAACGTCTCAG
Fig. 2c	Dusp1-qPCR-reverse	AACACTCTCCCTCCAGCATC
Fig. 2c	Tet1-qPCR-forward	ACACAGTGGTGCTAATGCAG
Fig. 2c	Tet1-qRT-reverse	AGCATGAACGGGAGAATCGG
Fig. 5d	Chr3:50212643_2761-5hmc-forward	TCTGTATTCAGAAGTATTAAGTCAG CA
Fig. 5d	Chr3:50212643_2761-5hmc-reverse	TTATCTGCATGAATGAGAATACGG
Fig. 5d	Chr9:48531283_1484-5hmc-forward	TCTAACCCCTACTCCCAGACC
Fig. 5d	Chr9:48531283_1484-5hmc-reverser	GTACTCCAAGGGCAGCAGAG
Fig. 5d	Ch12:28025035_5184-5hmc-forward	AAAGGGAACAATCTTGCTGTG
Fig. 5d	Ch12:28025035_5184-5hmc-reverse	AAGAAAAACAAAAACAAGAAGAG AAA
Fig. 5f	Slc7a11-qPCR-forward	CCCAGATATGCATCGTCCTT
Fig. 5f	Slc7a11-qPCR-reverse	CGTCTGAACCACTTGGGTTT
Fig. 5f	Mtr-qPCR-forward	CATCCAAGAGTGTGGTGGTG
Fig. 5f	Mtr-qPCR-reverse	ATAAACGTGGGCTTCACTGG
Fig. 5f	Sox11-qPCR-forward	TCATGTTCGACCTGAGCTTG
Fig. 5f	Sox11-qPCR-reverse	TAGTCGGGGAACTCGAAGTG
Fig. 5h	Egr1-5hmc-forward	ACAAGCTTCTCTCCATGCCT

Fig. 5h	Egr1-5hmc-reverse	CCACCAAGAAGCTTGCTGTT
Fig. 5h	Egr2-5hmc-forward	CGAGTGTGGGTGGTTGAAAT
Fig. 5h	Egr2-5hmc-reverse	CTCGCAACACTTCCCAACAG
Fig. 5h	Egr3-5hmc-forward	ACTTGGAGCCGAAATGAACAG
Fig. 5h	Egr3-5hmc-reverse	GAGGACGAGCATTCAGGACT
Fig. 5h	Npas4-5hmc-forward	TCCCGTTACTGGTTTGTAGAGA
Fig. 5h	Npas4-5hmc-reverse	CGTAGTGAGTTTCCTTGAATGGA
Fig. 5h	Fos-5hmc-forward	ACGTTTCCACTGTGTTGCAT
Fig. 5h	Fos-5hmc-reverse	AACTCAACCCTGTGCTCTCA
Fig. 5h	Arc-5hmc-forward	AACACAACCACTTCTCAGCC
Fig. 5h	Arc-5hmc-reverse	GGAGAGGTAAAGTGGGGTCC
