# Supplementary material for: Brain methylome remodeling selectively regulates neuronal activity genes linking to emotional behaviors in mice exposed to maternal immune activation
Source: Nat Commun. 2023 Nov 29;14:7829. doi: 10.1038/s41467-023-43497-4 (PMC10687003; doi:10.1038/s41467-023-43497-4)
Supplement: Supplementary file 3 — Reporting Summary [file 41467_2023_43497_MOESM3_ESM.pdf]

Corresponding author(s): Bing Yao and Jianfu Chen
Last updated by author(s): Sep 20, 2023

# Reporting Summary

Nature Portfolio wishes to improve the reproducibility of the work that we publish. This form provides structure for consistency and transparency in reporting. For further information on Nature Portfolio policies, see our Editorial Policies and the Editorial Policy Checklist.

## Statistics

For all statistical analyses, confirm that the following items are present in the figure legend, table legend, main text, or Methods section.

| n/a | Confirmed | |
|---|---|---|
| ☐ | ☒ | The exact sample size (*n*) for each experimental group/condition, given as a discrete number and unit of measurement |
| ☐ | ☒ | A statement on whether measurements were taken from distinct samples or whether the same sample was measured repeatedly |
| ☐ | ☒ | The statistical test(s) used AND whether they are one- or two-sided<br>*Only common tests should be described solely by name; describe more complex techniques in the Methods section.* |
| ☒ | ☐ | A description of all covariates tested |
| ☐ | ☒ | A description of any assumptions or corrections, such as tests of normality and adjustment for multiple comparisons |
| ☐ | ☒ | A full description of the statistical parameters including central tendency (e.g. means) or other basic estimates (e.g. regression coefficient) AND variation (e.g. standard deviation) or associated estimates of uncertainty (e.g. confidence intervals) |
| ☐ | ☒ | For null hypothesis testing, the test statistic (e.g. *F*, *t*, *r*) with confidence intervals, effect sizes, degrees of freedom and *P* value noted<br>*Give P values as exact values whenever suitable.* |
| ☒ | ☐ | For Bayesian analysis, information on the choice of priors and Markov chain Monte Carlo settings |
| ☒ | ☐ | For hierarchical and complex designs, identification of the appropriate level for tests and full reporting of outcomes |
| ☒ | ☐ | Estimates of effect sizes (e.g. Cohen's *d*, Pearson's *r*), indicating how they were calculated |

*Our web collection on statistics for biologists contains articles on many of the points above.*

## Software and code

Policy information about availability of computer code

| | |
|---|---|
| Data collection | Real-Time PCR: QuantStudio 3;<br>Bulk RNA-seq: Illumina Hi-seq system;<br>5hmC-seq for cortex: Illumina Hi-seq system;<br>5hmC-seq and MeDIP-seq for NeuN+ and NeuN-: Illumina NovaSeq X plus system;<br>snRNA-seq and Em-seq for cortex: Illumina NovaSeq 6000 system;<br>Flow cytometry: BD FACSDiva v9.0;<br>Fluorescent images: Leica Application Suite (LAS) X Navigator software;<br>ELISA: SoftMAX Pro 7.1;<br>Behaviral tests: SMART 3.0. |
| Data analysis | Image processing and analysis: ImageJ v1.53s<br>Statistical analyses: GraphPad Prism v9.0.0<br>ELISA data acquisition and analysis: SoftMAX Pro 7.1<br>Animal behavior tracking and analyses: SMART 3.0<br>Demultiplexing and alignment of sequencing reads to the mouse transcriptome: CellRanger v3.1.0<br>Bioinformatic analyses of snRNA-seq: Seurat v4.0<br>Bioinformatic analyses: R v3.6.0<br>5mC data mapping and methylation calling: Bismark v.0.17.0<br>RNA-seq sequence alignment: TopHat v.2.1.0<br>All the sequencing data filter and trimming: Trim Galore v0.4.1<br>5hmC-seq sequence alignment: Bowtie2 v2.3.5.1 |

5hmC concatenated reads calculation: Ngsplot v2.63
Annotation analyses of genomic features: HOMER v4.11.1
5mC methylation identification and characterization: RADmeth v1.0
CHROHMM annotation: CHROHMM v1.24, BEDtools v2.28.0
Differential analyses for genes and repetitive elements: TEtranscripts v2.2.3

For manuscripts utilizing custom algorithms or software that are central to the research but not yet described in published literature, software must be made available to editors and reviewers. We strongly encourage code deposition in a community repository (e.g. GitHub). See the Nature Portfolio guidelines for submitting code & software for further information.

## Data

Policy information about availability of data

All manuscripts must include a data availability statement. This statement should provide the following information, where applicable:

- Accession codes, unique identifiers, or web links for publicly available datasets
- A description of any restrictions on data availability
- For clinical datasets or third party data, please ensure that the statement adheres to our policy

5hmC and RNAseq data have been deposited in the NCBI Gene Expression Omnibus (GEO) database under accession number GSE163268 (https://www.ncbi.nlm.nih.gov/geo/query/acc.cgi?acc=GSE163268).
The 5mC and snRNA-Seq data have been in deposited the GEO database under accession number GSE164783 (https://www.ncbi.xyz/geo/query/acc.cgi?acc=GSE164783).
The MeDIP-seq and 5hmC-seq for neuron and non-neuron cells have been deposited into the GEO database under accession number GSE240417 (https://www.ncbi.nlm.nih.gov/geo/query/acc.cgi?acc=GSE240417).

## Research involving human participants, their data, or biological material

Policy information about studies with human participants or human data. See also policy information about sex, gender (identity/presentation), and sexual orientation and race, ethnicity and racism.

| | |
|---|---|
| Reporting on sex and gender | not available |
| Reporting on race, ethnicity, or other socially relevant groupings | not available |
| Population characteristics | not available |
| Recruitment | Not available |
| Ethics oversight | Not available |

Note that full information on the approval of the study protocol must also be provided in the manuscript.

# Field-specific reporting

Please select the one below that is the best fit for your research. If you are not sure, read the appropriate sections before making your selection.

☒ Life sciences ☐ Behavioural & social sciences ☐ Ecological, evolutionary & environmental sciences

For a reference copy of the document with all sections, see nature.com/documents/nr-reporting-summary-flat.pdf

# Life sciences study design

All studies must disclose on these points even when the disclosure is negative.

| | |
|---|---|
| Sample size | No statistical methods were used to predetermined sample sizes. Our sample sizes were determined on the basis of our published studies (PMID: 34232545, 37228107).The sample size for each experiment is indicated in the figure legends. |
| Data exclusions | For snRNA-seq analyses, poor quality cells with less than 200 genes and mitochondrial gene percentage >50 were removed in this study. |
| Replication | All experiments were performed in at least three biological replicates and all results presented in this manuscript were reliably reproducible, which are representative of at least 3 independent experiments. Specific sample sizes and statistical analysis and significances of the results are indicated in the figure legends. |
| Randomization | Pregnant female mice were randomly selected for mock and treatment groups in this study. Age and gender-matched offspring mice were used across groups. |
| Blinding | All the bioinformatic parts were performed with identical pipeline for each experiment, therefore blinding not relevant for bioinformatic analyses;<br>Behavioral tests were performed and analyzed by experimenters who were blind to different treatment groups. |

For Immunofluorescent staining, ELISA, real-time qPCR and flow cytometry , investigators were not blinded during data acquisition and data analysis, because the consistent data acquisition and data analysis settings were applied to all samples of the experiments.

# Reporting for specific materials, systems and methods

We require information from authors about some types of materials, experimental systems and methods used in many studies. Here, indicate whether each material, system or method listed is relevant to your study. If you are not sure if a list item applies to your research, read the appropriate section before selecting a response.

## Materials & experimental systems

| n/a | Involved in the study |
|---|---|
| ☐ | ☒ Antibodies |
| ☐ | ☒ Eukaryotic cell lines |
| ☒ | ☐ Palaeontology and archaeology |
| ☐ | ☒ Animals and other organisms |
| ☒ | ☐ Clinical data |
| ☒ | ☐ Dual use research of concern |
| ☒ | ☐ Plants |

## Methods

| n/a | Involved in the study |
|---|---|
| ☒ | ☐ ChIP-seq |
| ☐ | ☒ Flow cytometry |
| ☒ | ☐ MRI-based neuroimaging |

## Antibodies

Antibodies used

Rabbit anti-c-Fos (Cell Signaling Technology, Cat# 2250, Clone: 9F6, 1:400)
Rabbit anti-Egr1 (Cell Signaling Technology, Cat# 4153, Clone: 15F7, 1:200)
Rabbit anti-Npas4 (Novus Biologicals, NBP2-47252, 1:200)
Mouse anti-NeuN antibody (EMD Millipore, Cat# MAB377, Clone: A60, 1:400)
FITC anti-NeuN antibody (Abcam, Cat#ab223994, Clone: EPR12763, 1:150)
FITC-rabbit IgG isotype control (Abcam, Cat#ab223339, Clone: EPR25A, 1:150)
Goat anti-Rabbit secondary antibodies (A-11011, Thermo Fisher Scientific, 1:200)
Goat anti-Mouse IgG1 Cross-Adsorbed Secondary Antibody (A-21240, 1:200)

Validation

Rabbit anti-c-Fos (Cell Signaling Technology, Cat# 2250, Clone: 9F6)
Vendor information:https://www.cellsignal.com/products/primary-antibodies/c-fos-9f6-rabbit-mab/2250
Application: immunofluorescence (1:200, validated by PMID:33459594; 1:500 validated by PMID: 32369018, 27683910, 27008987; 1:100 validated by PMID: 32859919)

Rabbit anti-Egr1 (Cell Signaling Technology, Cat# 4153, Clone: 15F7)
Vendor information:https://www.cellsignal.com/products/primary-antibodies/egr1-15f7-rabbit-mab/4153
Application: immunofluorescence (1:1000, validated by PMID: 32369018; 1:300 validated by PMID: 32210755)

Rabbit anti-Npas4 (Novus Biologicals, NBP2-47252)
Vendor information:https://www.novusbio.com/products/npas4-antibody_nbp2-47252
Application: immunofluorescence (1:2000, validated by PMID: 23598442; 1:100 validated by PMID: 21312224)

Mouse anti-NeuN antibody (EMD Millipore, Cat# MAB377, Clone: A60)
Vendor information:
Application: immunofluorescence (1:200, validated by PMID: 2606710;4 1:500, validated by PMID: 32210755; 1:1000 validated by PMID: 26373451, 26140685; 1:1500 validated by PMID: 26194807)

FITC anti-NeuN antibody (Abcam, Cat# ab223994, Clone: EPR12763)
Vendor information:https://www.abcam.com/products/primary-antibodies/fitc-neun-antibody-epr12763-neuronal-marker-ab223994.html
Application: immunofluorescence (1:100, validated by PMID: PMID: 33300254,32937134); flow cytometry (1:50, validated by correspondent manufacturer)

FITC-rabbit IgG isotype control (Abcam, Cat# ab223339, Clone: EPR25A)
Vendor information:https://www.abcam.com/products/primary-antibodies/fitc-rabbit-igg-monoclonal-epr25a-isotype-control-ab223339.html
Application: immunofluorescence (1:100, validated by PMID: 31651935, 31425004); flow cytometry (1:50, validated by correspondent manufacturer)

Goat anti-Rabbit secondary antibodies (Cat# A-11011, Thermo Fisher Scientific)
Vendor information:https://www.thermofisher.com/antibody/product/Goat-anti-Rabbit-IgG-H-L-Cross-Adsorbed-Secondary-Antibody-Polyclonal/A-11011
Application: immunofluorescence (1:250, validated by PMID: 37581939; 1:500, validated by PMID: 32210755, 37783895; 1:1000, validated by PMID: 37813838)

Goat anti-Mouse IgG1 Cross-Adsorbed Secondary Antibody (Cat# A-21240, Thermo Fisher Scientific)
Vendor information:https://www.thermofisher.com/antibody/product/Goat-anti-Mouse-IgG1-Cross-Adsorbed-Secondary-Antibody-Polyclonal/A-21240

Application: immunofluorescence (1:400, validated by PMID: 37748809; 1:800, validated by PMID: 37501197; 1:1000, validated by PMID: 37694145, 37080203)

## Eukaryotic cell lines

Policy information about cell lines and Sex and Gender in Research

| | |
|---|---|
| Cell line source(s) | Vero cell line was obtained from the American Tissue Culture Collection (ATCC). The Vero cell was used for Zika virus production at a multiplicity of infection (MOI) of 0.01. |
| Authentication | We did not perform addtional authentication of this cell line. |
| Mycoplasma contamination | The cell line was tested negative mycoplasma. |
| Commonly misidentified lines (See ICLAC register) | No commonly misidentified cell lines were used. |

## Animals and other research organisms

Policy information about studies involving animals; ARRIVE guidelines recommended for reporting animal research, and Sex and Gender in Research

| | |
|---|---|
| Laboratory animals | Wild-type C57BL/6 mice (stock number: B6-F, 8-12 weeks old) was obtained from the Taconic Biosciences. The mice were kept under specific pathogen-free (SPF) condition in a 12 h light/12 h dark cycle (7:00 a.m. to 7:00 p.m.) at 20-24 °C and contolled humidity (40-60%, usually around 50%) with free access to food and water.<br>Timed pregnant female mice (8-12 weeks old) were obtained by mating with males and the presence of seminal plugs was considered embryonic day (E) 0.5. Pregnant female mice received intravenous injection of ZIKV or PBS at E12.5 and were returned to home cages.<br>Behavioral tests of ZIKV or mock offspring mice were performed at age of 8-12 weeks. Brain samples were collected at postnatal day 21 for library construction for omics analysis. |
| Wild animals | This study did not invovle wild animals. |
| Reporting on sex | Both male and female mice were used for hehavioral tests. The results were analyzed both separately and combined.<br>Male mice were used for Bulk RNA-seq, snRNA-seq, 5hmC and 5mC profiling to avoid additional variation introduced by estrous cycle of female mice. |
| Field-collected samples | This study did not involve samples collected from the field. |
| Ethics oversight | All animal procedure were approved by the Department of Animal Resources and Institute Animal Care and Use Committee of the University of Southern California (protocol 20719). |

Note that full information on the approval of the study protocol must also be provided in the manuscript.

## Flow Cytometry

### Plots

Confirm that:

☒ The axis labels state the marker and fluorochrome used (e.g. CD4-FITC).

☒ The axis scales are clearly visible. Include numbers along axes only for bottom left plot of group (a 'group' is an analysis of identical markers).

☐ All plots are contour plots with outliers or pseudocolor plots.

☒ A numerical value for number of cells or percentage (with statistics) is provided.

### Methodology

| | |
|---|---|
| Sample preparation | The neuron/non-neuoron cells were isolated as previously described (Brewer and Torricelli, 2007) with minor modifications. Briefly, The anesthesized mice were perfused with ice-cold 30 ml DPBS and the brains were collected. The bilateral prefrontal cortices were raply dissected in the ice-old HABG buffer and sliced using MacIlwain tissue chopper with thickness of 500 µm. The slices were digested in 0.2% papain in HA buffer for 30 min at 30 °C and then triturated using fire-polished Pateur pipette. Cell suspension was applied to Optiprep density gradient and was centrifuged at 1900 rpm for 15 min at 22 °C. Neuons were collected from Fraction 3 and non-neuron cells were collected from Fraction 1 and 4. Fraction 2 was discarded. |
| Instrument | BD LSRII |
| Software | BD FACSDiva v9.0 |
| Cell population abundance | The purity of neurons isolated from Optiprep density gradient-based : approximate 86%; |

| | |
|---|---|
| Cell population abundance | The purity of non-neuronal cells isolated from Optiprep density gradient-based : approximate 85.5%; |
| Gating strategy | Gates were performed separating neurons and non-neuronal cells by FSC/SSC and expression of NeuN. |

☐ Tick this box to confirm that a figure exemplifying the gating strategy is provided in the Supplementary Information.
